# Galactosaminogalactan (GAG) and its multiple roles in *Aspergillus* pathogenesis

**DOI:** 10.1080/21505594.2019.1568174

**Published:** 2019-01-22

**Authors:** Cornelia Speth, Günter Rambach, Cornelia Lass-Flörl, P. Lynne Howell, Donald C. Sheppard

**Affiliations:** a Division of Hygiene and Medical Microbiology, Medical University of Innsbruck, Innsbruck, Austria; b Christian Doppler Laboratory for Invasive Fungal Infections, Innsbruck, Austria; c Program in Molecular Medicine, The Hospital for Sick Children, Toronto, Canada; d Department of Biochemistry, University of Toronto, Toronto, Canada; e Departments of Medicine and of Microbiology and Immunology, McGill University, Montréal, Canada; f Infectious Diseases and Immunity in Global Health Program, Research Institute of the McGill University Health Centre, Montréal, Canada

**Keywords:** Galactosaminogalactan, aspergillosis, host pathogen interactions, virulence factor

## Abstract

*Aspergillus* spp and particularly the species *Aspergillus fumigatus* are the causative agents of invasive aspergillosis, a progressive necrotizing pneumonia that occurs in immunocompromised patients. The limited efficacy of currently available antifungals has led to interest in a better understanding of the molecular mechanisms underlying the pathogenesis of invasive aspergillosis in order to identify new therapeutic targets for this devastating disease. The *Aspergillus* exopolysaccharide galactosaminogalactan (GAG) plays an important role in the pathogenesis of experimental invasive aspergillosis. The present review article summarizes our current understanding of GAG composition and synthesis and the molecular mechanisms whereby GAG promotes virulence. Promising directions for future research and the prospect of GAG as both a therapy and therapeutic target are reviewed.

## Introduction

In order to cause pulmonary infection, microorganisms must both adhere to host cells, adapt to the natural environment imposed by the pulmonary environment and evade immune responses. One strategy used by the mold *Aspergillus fumigatus* to establish and maintain pulmonary infection is the production of biofilms during invasive infection in immunocompromised individuals and airway infection in patients with chronic lung disease [1]. Biofilms consist of stratified communities of organisms growing within a thick slime-like matrix of polysaccharides, proteins, lipids and nucleic acids that protect fungi from immune mediated killing and enhance resistance to antifungal agents [2,3]. Recent studies have established a key role for the exopolysaccharide galactosaminogalactan (GAG) in both the formation of *A. fumigatus* biofilms and in modulating the immune response during invasive infection.

GAG is a heteropolysaccharide composed of α-1,4 linked galactose, N-acetyl galactosamine (GalNAc) and galactosamine (GalN) [4–6] that is secreted by actively growing hyphae. GAG binds to the surface of these hyphae, resulting in a polysaccharide sheath that covers the growing organism and forms an extracellular matrix between hyphae [5]. GAG is expressed during chronic and invasive infection, and the production of cell wall GAG correlates with the intrinsic virulence of *Aspergillus* species [7]. Strains deficient in GAG do not form biofilms and are less virulent in mouse models of invasive aspergillosis (IA) [5]. Herein, we review our current understanding of the mechanisms underlying the synthesis of GAG, its role in the pathogenesis of invasive aspergillosis, and the current status of efforts to develop therapeutics targeting this important exopolysaccharide [5].

## GAG biosynthesis

**Figure 1.**
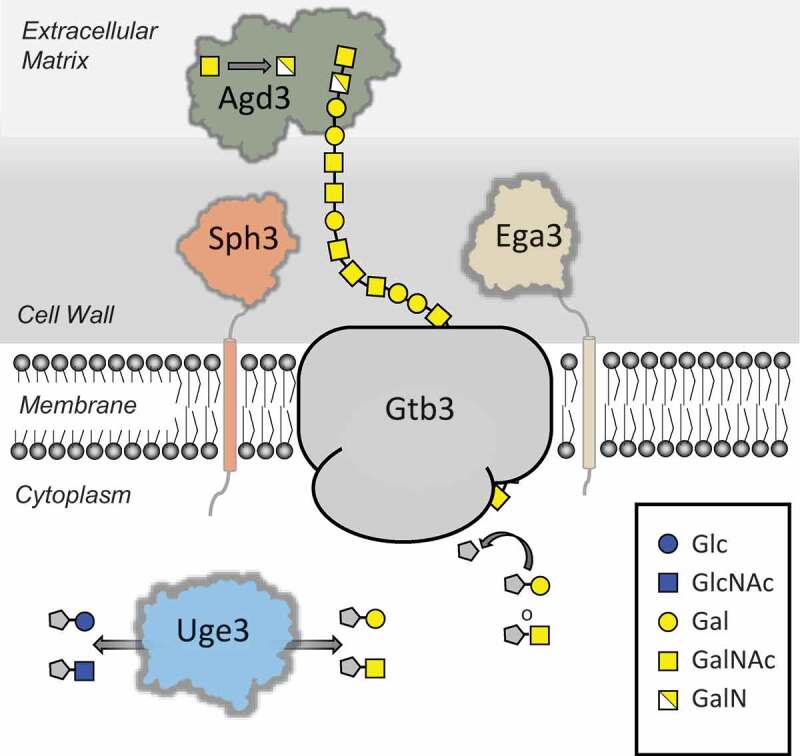
GAG polysaccharide biosynthetic pathway. Schematic representation of the proteins involved in (i) production of the GAG activate sugar nucleotide precursors (Uge3), (ii) polymerization and transport across the membrane (Gtb3), and (iii) hydrolysis (Sph3/Ega3) and deacetylation (Adg3) of the mature polymer. Abbreviations: Glc, Glucose; GlcNAc, N-acetylglucosamine; Gal, Galactose, GalNAc, N-acetylglucosamine; GalN, Galactosamine. UDP is denoted by a grey pentagon.

The biosynthetic pathway governing GAG production was identified by comparative transcriptional analyzes of *A. fumigatus* regulatory mutants deficient in the production of GAG [5]. This approach identified a cluster of five co-regulated genes on chromosome 3 which are predicted to encode enzymes with carbohydrate synthetic or modifying capacity [8]. Through gene disruption as well as structural and biochemical studies, a model of the function of these enzymes in GAG biosynthesis has begun to emerge (Figure 1). Synthesis of GAG begins with the conversion of UDP-glucose and UDP-N-acetyl glucosamine into UDP-galactose and UDP-N-acetyl galactosamine through the action of the cytosolic glucose-4 epimerase Uge3 [5,6]. Linking of these sugars, and export into the extracellular space is hypothesized to be mediated by the glycosyl transferase Gtb3, although experimental verification of the role of this enzyme in GAG synthesis remains to be confirmed. GalNac sugars within the newly secreted polymer are then deacetylated by the secreted Agd3 deacetylase, rendering the polymer cationic [8]. Deacetylation is required for GAG adhesion to the surface of hyphae and biofilm formation [8]. Two other genes within the GAG biosynthetic cluster encode putative glycosyl hydrolases. The *sph3* gene product, Sph3, has been expressed and structurally and functionally characterized as a member of a novel glycosyl hydrolase class that is predicted to be anchored to the cell membrane of *A. fumigatus* [9]. The recombinant glycosyl hydrolase domain of Sph3 cleaves purified and hyphal-associated GAG, confirming the specificity of this protein for GAG [9]. The ∆*sph3* mutant is unable to produce GAG, suggesting that polymer cleavage is required for synthesis or export of the mature polymer [9]. The final gene in the cluster, *ega3*, is predicted to encode an endogalactosaminidase, however experimental confirmation of the function of this protein remains to be performed.

Although substantial progress has been made in elucidating the mechanisms underlying the synthesis of GAG, a number of questions remain unanswered. GAG contains regions of homopolymeric GalNAc and galactose as well as heteropolymeric GalNAC-galactose. It is not known if Gtb3 is able to synthesize all of these sugar combinations, or if the participation of other glycosyl transferases that are not encoded within the chromosome 3 cluster is required. It is also not known how the membrane-localized hydrolase Sph3, and potentially Ega3, contribute to synthesis of GAG if their primary function is to cleave the polymer. One hypothesis is that, like the Gel proteins of *A. fumigatus* [10], Sph3 not only cleaves GAG, but also exhibits transferase activity and links together cleaved GAG oligosaccharides. Alternately, these enzymes could play a role in controlling polymer length or release of the newly synthesized glycan for transport to the outer cell wall and biofilm matrix. Further work is required to distinguish among these possibilities.

A number of factors that govern the expression of GAG production have been identified. Early studies identified a link between developmental regulatory factors and GAG gene expression. Microarray studies demonstrated that GAG gene cluster expression is dependent on both MedA and StuA, factors that also regulate conidiation in *A. fumigatus* [5,11]. More recently, the SomA/PtaB complex, thought to be downstream of the cAMP/PKA pathway, has been found to play an important role in governing GAG-gene expression, in part via induction of MedA [12,13]. Interestingly, PtaB expression was found to regulate the expression of *uge3* and *agd3* specifically within the GAG gene cluster, suggesting the possibility that different stages of GAG synthesis may be independently regulated.

## GAG serology

GAG is antigenic, and GAG-specific antibodies are found in the majority of the human population, even in the absence of clinically apparent *Aspergillus* infection. Tests with blood bank sera revealed the presence of anti-GAG antibodies, mainly IgG2, in 40% of healthy donors and a comparable percentage of patients with invasive aspergillosis [4]. Similarly, *A. fumigatus*-infected and uninfected rats both were found to have high levels of anti-GAG antibodies [14]. These seroprevalence data and the lack of correlation of antibody titers with clinical aspergillosis, suggest that anti-GAG antibodies are not useful biomarker for invasive aspergillosis. This hypothesis is confirmed by the fact that infection with *Aspergillus* was not associated with a change in anti-GAG serum titers [4]. Furthermore, the high GAG seroprevalence suggests that these native anti-GAG antibodies do not mediate protection against invasive aspergillosis. Although it is not known why these antibodies do not protect against *A. fumigatus* infection, this observation may reflect impaired neutrophil and other effector function in immunocompromised patients, despite recognition of fungal hyphae by antibodies. Additionally, the epitopes bound by natural anti-GAG antibodies have not been defined, and it is possible that these antibodies bind to GalNAc or galactose-rich regions of GAG rather than deacetylated polymer, and as such do not interfere with GAG function. Studies to evaluate the effects of anti-GAG antibodies on *A. fumigatus* adhesion and virulence and the epitope specificity of these antibodies are required to test these hypotheses.

While the precise epitope characterization of circulating anti-GAG antibodies has yet to be performed, it has been speculated that the anti-GAG antibodies that are observed in healthy donors are a consequence of cross-reactivity of antibodies produced in response to other microbial glycans. In support of this hypothesis, cross-reactivity of anti-GAG antibodies with the *N*-glycans of *Campylobacter jejuni* cell surface glycoproteins has been reported [4]. Additionally, the PelA glycoside hydrolases from the bacterium *Pseudomonas aeruginosa* is able to bind and cleave GAG, suggesting GAG and the bacterial exopolysaccharide Pel may share structural similarities [15].

As an adhesive molecule, GAG has been reported to mediate interaction with a number of other host and microbial factors. Adherence and pull-down assays *in vitro* revealed that the host protein surfactant D binds purified *A. fumigatus* GAG as well as melanin and galactomannan [16]. Verification of SP-D binding to GAG on *A. fumigatus* hyphae has yet to be performed. In studies of fungal-bacterial interactions *in vitro, Pseudomonas aeruginosa* was found to bind purified GAG [17]. Studies using a GAG-deficient mutant of *A. fumigatus* confirmed that bacterial binding to hyphae was GAG-dependent [17]. The importance of this interaction in co-operative biofilm formation in vitro and in vivo remain to be elucidated.

## The role of GAG in host pathogen interactions

**Figure 2.**
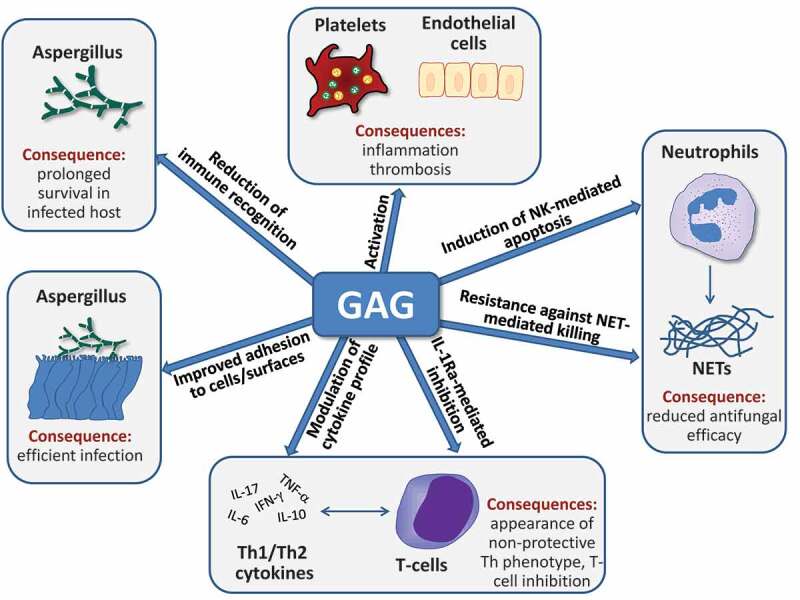
Multiple potential roles of GAG during fungal infection. GAG is required for efficient adherence of *A. fumigatus* hyphae to host cells and surfaces and protects the fungal cell wall from immune recognition. Furthermore, it can activate platelets and endothelial cells and thus contribute to thrombosis during the pathogenesis of fungal infection. GAG reduces the antifungal capacity of neutrophils by two mechanisms, the induction of apoptosis and the conference of resistance against neutrophil extracellular traps (NET). Finally, T-cell responses to GAG include altered cytokine production as a consequence of GAG-dependent IL-1 receptor antagonist (IL-1Ra) production, leading to reduced Th1 and Th17 cytokine production. For further details, see text.

GAG is both secreted and associated with the surface of *A. fumigatus* hyphae and is therefore at the frontline of interplay between *A. fumigatus* and the host. GAG plays a role in a wide range of host-pathogen interactions including adhesion to host cells, evasion and modulation of immune response and platelet activation. These different aspects of GAG are described in the following sections and also summarized in Figure 2.

Two experimental approaches have been taken to study the role of GAG in host-pathogen interactions: the study of mutants with altered GAG expression, and the use of purified fractions of soluble GAG. Although both of these approaches have advanced our understanding of this important fungal exopolysaccharide, it is important to recognize the limitations of each approach. Mutants with altered cell wall polysaccharide expression can exhibit alterations in expression of other cell wall glycans as a consequence of substrate flux or as a compensatory response to changes in cell wall integrity. For example, mutations in the *uge5, ugm1* genes within the *A. fumigatus* galactomannan synthesis pathway result in increased production of GAG [6,18]. Conversely, as GAG is a highly insoluble polymer, studies of direct polysaccharide interactions with host cells have largely relied on a soluble fraction of GAG, which is enriched in galactose and lacks deacetylated GalNAc (GalN) [4]. It is not known if this fraction of GAG induces similar host responses to the native deacetylated heteropolymer. Correlation of the results of both types of studies is therefore critical to understand the function of GAG in host-pathogen interactions. Throughout this review, the experimental approach used is outlined in order to aid in interpreting the findings.

### GAG as an adhesin

Adherence and subsequent invasion of host cells is an important step in the pathogenesis of invasive aspergillosis. Multiple lines of evidence suggest GAG plays a major role in mediating adherence of hyphae to host cells and other substrates [19]. The GAG-deficient ∆*uge3* and ∆*medA* mutants exhibit markedly reduced adherence to the A549 pulmonary epithelial cell line, and a wide variety of abiotic substrates [5,13]. Purified GAG adheres directly to A549 epithelial cells and enhances adhesion of hyphae of the ∆*uge3* mutant to surfaces [5]. These findings were confirmed by atomic force microscopy studies of epithelial cell and hydrophobic surface interactions with the ∆*uge3* mutant [20]. Finally, GAG-overexpressing ∆*uge5* and ∆*ugm1* as a consequence of mutations in the galactofuranose synthesis pathway exhibit increased adherence to a range of host cells and surfaces [6,18,21,22].

Deacetylation of GAG by Agd3 is required not only for the adherence of GAG to the surface of hyphae, but also for GAG-mediated fungal adhesion to surfaces [8]. Deacetylation renders GAG polycationic, and therefore adhesive to anionic surfaces including the hyphal cell wall, plastic, and host cell membranes. As a result, the deacetylase-deficient ∆*agd3* mutant phenocopies the adhesion and virulence defects of the GAG-deficient ∆*uge3* mutant *in vitro* and *in vivo* [8].

### GAG interference with immune recognition

Cell wall β-1,3 glucans are important fungal pathogen-associated molecular patterns (PAMPs) that are recognized by the C-type lectin dectin-1 during infection. Studies of β-1,3 glucan exposure in *A. fumigatus* have demonstrated that these glycans are concealed in conidia by hydrophobins and only exposed as conidia swell and begin to germinate [23]. Upon germination however, β-1,3 glucan exposure declines due to production of hyphal-associated GAG, thus impairing dectin-1 recognition of hyphae [5]. Consequently, loss of GAG production in the Δ*uge3* mutant is associated with increased β-1,3 glucan exposure as demonstrated by lectin staining, scanning electron microscopy and atomic force microscopy [5,20,24]. Increased β-1,3 glucan exposure on GAG-deficient *A. fumigatus* hyphae is associated with increased leukocyte recruitment during pulmonary infection, and enhanced binding of dectin-1 to the surface of hyphae leading to higher levels of inflammatory cytokine production by dendritic cells *in vitro* [5]. Similar findings were reported in a study of the interactions of *Aspergillus nidulans* with peripheral blood mononuclear cells (PBMCs) from patients with chronic granulomatous diseases [24]. *A. nidulans* was found to produce less cell-wall associated GAG and induced higher levels of IL-1β secretion as compared to *A. fumigatus* [24]. Overexpression of GAG in *A. nidulans* resulted in lower levels of IL-1β secretion by PBMCs, confirming a direct link between GAG and a reduction of host inflammatory responses. GAG also likely plays a role in in concealing other cell wall PAMPs in addition to β-1,3 glucan, however this has not yet been studied.

### GAG and neutrophil interactions

Neutrophils are key effectors of the innate immune response against *Aspergillus* and are able to rapidly infiltrate into infected tissue to kill fungal cells. Reduced numbers or impaired function of neutrophils underlie the susceptibility of most patients to invasive aspergillosis. Both secreted and cell wall-associated GAG interfere with neutrophil-mediated immunity to *A. fumigatus*.

GAG has been implicated in the induction of neutrophil apoptosis. Whole blood samples treated with a purified soluble fraction of GAG were found to have higher numbers of apoptotic neutrophils [4]. Soluble GAG triggers neutrophils to produce reactive oxygen species (ROS), which in turn increase the expression of MHC class I chain-related molecule A (MIC-A) on the surface of neutrophils. Neutrophilic MIC-A enables the interaction with natural killer (NK) cells by binding to the NKG2D receptor on the NK cells. This process induces NK cell activation and triggers a Fas-dependent apoptosis signal to the neutrophils via the caspase-8 pathway [25].

The role of GAG in neutrophil apoptosis has not been confirmed *in vivo*. However, intratracheal treatment of mice with a purified soluble fraction of GAG during *A. fumigatus* infection resulted in reduced numbers of pulmonary neutrophils [4,26]. Similarly, higher numbers of neutrophils were observed surrounding fungal lesions during infection with the GAG-deficient Δ*uge3* mutant as compared to wild-type *A. fumigatus* [5]. Further studies are required to determine if these observations are due to GAG-induced neutrophil apoptosis, or other GAG-dependent effects on neutrophil recruitment such as the induction of IL-1 receptor antagonist (IL-1Ra) expression, or cloaking of cell wall PAMPs.

Studies of GAG production by other *Aspergillus* species have revealed that GAG plays a role in mediating resistance to extracellular killing by neutrophil extracellular traps (NETs). While *A. fumigatus* is not affected by NETS, these structures are able to kill the non-pathogenic species *Aspergillus nidulans* [27]. Unlike *A. fumigatus, A. nidulans* produces GalNAc-poor GAG that adheres poorly to the hyphal cell wall [7]. This difference in GAG composition of *A. nidulans* is due to low levels of expression of the *uge3* homolog, *ugeB* [7]. Overexpression of *ugeB*, or heterologous expression of *A. fumigatus uge3* in *A. nidulans* increases both the GalNAc content of GAG and the amount of GAG adherent to the hyphal cell wall [7]. This change in GAG composition and expression renders *A. nidulans* as resistant as *A. fumigatus* to NET-mediated killing, and increases the virulence of this species in a mouse model of invasive aspergillosis [7]. It has been hypothesized that GAG-mediated resistance to NETS is a consequence of electrostatic repulsion of cationic antimicrobial peptides within NETS by cationic GAG on the surface of hyphae, however this hypothesis awaits experimental validation.

### GAG, cytokines and T-cell response

GAG also exerts immunomodulatory effects through modulation of cytokine production and T-cell responses. Intranasal treatment with a purified soluble fraction of GAG significantly altered pulmonary cytokine production in a mouse model of invasive aspergillosis, with reduced mRNA levels of IFN-γ and IL-10 and higher levels of TNF-α, IL-6 and IL-17 [4]. This cytokine pattern downregulates the Th1-response and favors proliferation of the Th2 cell lineage, an immune response that is non-protective and promotes fungal growth [4,14,28]. Similarly, incubation of murine bone marrow-derived dendritic cells (DCs) with GAG-deficient Δ*uge3* hyphae resulted in the production of higher protein levels of pro-inflammatory cytokines (e.g. TNF-α, IL-6, MIP-1α) and lower levels of anti-inflammatory cytokines (IL-10) as compared to cells stimulated with GAG-producing wild-type *A. fumigatus* hyphae [5]. However, this increased pro-inflammatory cytokine production may also reflect unmasking of cell wall β-1,3 glucans in the absence of GAG, as treatment with soluble dectin-1 abrogated the increase in TNF-α protein production by DCs exposed to GAG-deficient Δ*uge3* hyphae [5].

Studies with human peripheral blood mononuclear cells (PBMCs) add further evidence that secreted GAG modulates cytokine production and T helper cell responses. Treatment with a purified, soluble fraction of GAG was found to downregulate PBMC protein expression of the cytokines IL-17 and IL-22 [26]. Detailed studies to elucidate the mechanism of this cytokine inhibition revealed that purified GAG stimulates the secretion of IL-1 receptor antagonist (IL-1Ra) by human PBMCs. IL-1Ra blocks the IL-1R type I receptor and thus suppresses IL-17 and IL-22 protein expression [14,26]. Intranasal treatment of mice with a purified soluble fraction of GAG led to increased production of IL-1Ra and higher pulmonary fungal burden, confirming the importance of GAG-induced IL-1Ra secretion *in vivo*. Finally, as noted above, induction of IL-1β secretion by PBMCs in response to *A. nidulans* infection was inversely proportional to GAG expression, although no effects on IL-1Ra production were observed in this system [24].

### GAG as activator of platelets and endothelium

In contrast to the multiple roles GAG plays in suppressing or evading immune responses, GAG is a direct activator of platelets. Platelets fulfil a dual role in homeostasis since they are a central part of the coagulation system and they possess a variety of immunological competences [29]. Although they are not professional immune cells such as neutrophils, they can exert a number of antimicrobial functions to either directly attack pathogens or to support the complex network of both adaptive and innate immune weapons [29,30]. Particularly their large number in the bloodstream can be supposed to outweigh any limitation in immune capacities.

A key first step for the participation of platelets in the innate immune response is activation with fusion of the internal alpha and dense granules with the membrane and release of their effector compounds (e.g. antimicrobial peptides, cytokines, chemokines) [29]. *Aspergillus fumigatus* induces contact-dependent platelet activation and degranulation [31,32]. Detailed analysis of the fungal surface structures involved in this process led to the identification of GAG as the key trigger of platelet activation [32]. Incubation of platelets with a purified soluble fraction of GAG stimulated release of alpha and dense granules content, alteration of the platelet membrane, and interaction of platelets with foreign particles [32].

Recently, we have found that soluble native GAG produced by *A. fumigatus* hyphae can directly activate platelets (Deshmukh et al, submitted). Secreted GAG bound to the surface of platelets and triggered platelet stimulation in a dose- and time-dependent manner as measured by CD62P exposure. Thus, *A. fumigatus* can activate platelets not only locally by direct contact with fungal hyphae, but also at a distance through interaction with secreted and circulating GAG molecules.

Taken as a whole, these *in vitro* studies suggest that GAG-induced platelet activation and degranulation may contribute substantially to the pathogenesis of invasive aspergillosis by inducing platelet aggregation and thrombosis, two hallmarks of pulmonary fungal infections [33]. GAG-induced thrombosis might not only be the result of platelet activation but could also reflect the direct effect of GAG on endothelial cells. A recent study revealed that both a soluble fraction of GAG and the GAG-overproducing ∆*ugm1 A. fumigatus* mutant induce a prothrombotic and hyperadhesive phenotype in endothelial cells including the upregulation of proinflammatory TNF-α protein and tissue factor mRNA [21,22]. However, given the multiple effects of GAG on immune effector cells, defining the role of GAG-platelet and GAG-endothelium interactions in the pathogenesis of invasive aspergillosis is challenging and will require the combined study of both GAG-deficient strains of *A. fumigatus* and platelet-deficient animals.

## The future of GAG: Therapeutic target or novel therapeutic

Given the multiple roles that GAG plays in the pathogenesis of invasive aspergillosis, therapeutics targeting this polysaccharide have the potential to attenuate virulence and even increase susceptibility to antifungal therapy. The most advanced of these approaches is the use of glycoside hydrolases to degrade GAG. Treatment of hyphae with the recombinant hydrolase domain of the GAG biosynthetic protein Sph3 degrades cell wall- and biofilm matrix-associated GAG; consequences are the disruption of fungal biofilms, increased susceptibility to antifungal agents and diminished ability of hyphae to adhere to and damage host cells *in vitro* [15]. Intratracheal administration of Sph3 is well tolerated by neutropenic mice, and attenuates fungal virulence when given at the time of infection [15]. Determining the activity of Sph3 and other hydrolases in combination with antifungal agents represents an important future direction in assessing the potential of these agents to treat or prevent invasive aspergillosis.

The ability of GAG to mediate immunosuppressive effects has led to interest in the use of soluble GAG in the treatment of inflammatory diseases. In a proof of concept study, the effects of intratracheal treatment with purified GAG were evaluated in mouse models of two inflammatory conditions. In allergic bronchopulmonary aspergillosis (ABPA), GAG treatment reduced neutrophilic inflammation in association with a reduction in Th17 cell markers within draining lymph nodes [26]. In a mouse model of murine colitis models, GAG therapy also improved the inflammatory reaction and induced IL-1Ra and IL-10 production [26]. The effects of GAG on both inflammatory diseases were similar to those observed with IL-1Ra administration, suggesting that the immunosuppressive effects of purified GAG are largely mediated by induction of this cytokine.

## Conclusion

The fungal exopolysaccharide GAG plays an important role in the pathogenesis of invasive aspergillosis. Although recent studies have begun to shed light on the biosynthesis and role of this glycan in pathogenesis, many questions remain to be answered. The potential of GAG both as a therapy and therapeutic target has been demonstrated in key proof of concept studies, and future studies to define the utility of these therapies are ongoing.
